# Spontaneous massive hemothorax due to inferior phrenic artery pseudoaneurysm: endovascular treatment approach

**DOI:** 10.1590/1677-5449.202501272

**Published:** 2026-01-09

**Authors:** Resham Singh, Annoushka Daniel, Naveen Kumar

**Affiliations:** 1 Vardhman Mahavir Medical College & Safdarjung Hospital, New Delhi, India.

**Keywords:** hemothorax, pseudoaneurysm, endovascular, embolization, hemotórax, pseudoaneurisma, endovascular, embolização

## Abstract

Inferior phrenic artery (IPA) pseudoaneurysms are highly uncommon and result from trauma, pancreatitis, or iatrogenic injury. However, rupture can lead to catastrophic hemothorax. The vessel’s deep location and non-specific symptoms make early identification a difficult task. We report a case of a 22-year-old woman who was undergoing antitubercular therapy and presented with the onset of acute dyspnea after a five-month history of wheezing and hemoptysis. Computed tomography angiography revealed a pseudoaneurysm originating from the left IPA and a massive left hemothorax. This pseudoaneurysm sac and the descending branch of the IPA were successfully embolized with a glue: lipiodol mixture, followed by hemothorax evacuation. The patient is asymptomatic at 2-month follow-up. Endovascular embolization minimizes morbidity and enables rapid recovery when anatomy is favorable. This report emphasizes the importance of cross-sectional imaging in the early diagnosis and treatment planning of atypical hemothorax presentations.

## Resumo

O pseudoaneurisma da artéria frênica inferior (AFI) é extremamente raro e resulta de trauma, pancreatite ou lesão iatrogênica. No entanto, sua ruptura pode levar a um hemotórax catastrófico. A localização profunda do vaso e os sintomas inespecíficos tornam a identificação precoce um desafio. Relatamos o caso de uma mulher de 22 anos que estava recebendo tratamento para tuberculose quando apresentou dispneia aguda após 5 meses de história de sibilos e hemoptise. A angiografia por tomografia computadorizada revelou um pseudoaneurisma originado na AFI esquerda e um hemotórax maciço à esquerda. Foi realizada embolização do “saco” do pseudoaneurisma e do ramo descendente da AFI com uma mistura de cola e lipiodol e, em seguida, evacuação do hemotórax. A paciente encontrava-se assintomática após 2 meses de acompanhamento. A embolização endovascular minimiza a morbidade e permite uma recuperação rápida quando a anatomia é favorável. Esse relato enfatiza a importância das imagens transversais no diagnóstico precoce e no planejamento do tratamento de apresentações atípicas de hemotórax.

## INTRODUCTION

The inferior phrenic arteries (IPA) are diaphragmatic branches that are typically paired and originate from the celiac trunk or abdominal aorta.^1^ Cross-sectional imaging is indispensable due to the fact that IPA pathology is frequently concealed on chest X-rays due to the vessel’s retrocrural course and diminutive caliber. Pseudoaneurysms of these vessels are exceptionally rare, but can result in hemothorax, abdominal hemorrhage, or shock by rupture.^2^ Management of these patients was previously dominated by open surgical repair; however, current data suggest that endovascular embolization should be the initial treatment.^1-3^ We present a case of IPA pseudoaneurysm that manifested as massive hemothorax. The pseudoaneurysm was successfully embolized endovascularly and hemothorax evacuation was performed.

## CASE REPORT

**Figure 1:**
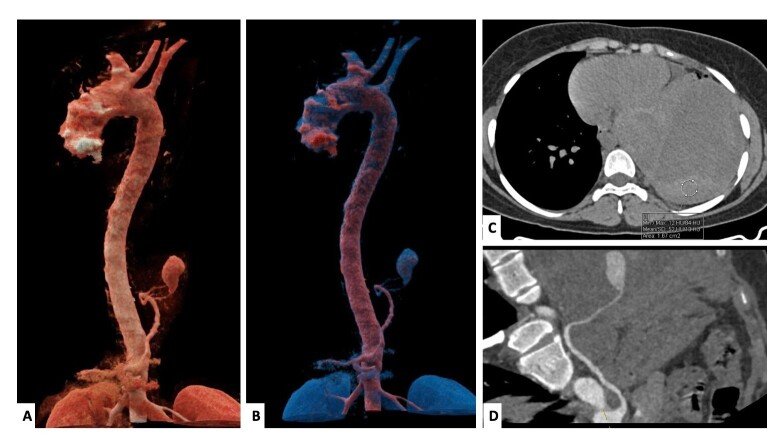
Cinematic volume rendered tomography images (Panel A, B) and non-contrast axial image of thorax (Panel C) and curved planar reformatted (CPR) image (Panel D) show a large hyperdense (HU 52) hematoma in the left hemithorax with resultant massive hemothorax and underlying lung collapse. The left inferior phrenic artery (LIPA) is seen arising from the ostioproximal celiac artery, with a large pseudoaneurysm arising from it just above the diaphragm, with a large surrounding hematoma.

A 22-year-old female presented with recent exacerbation of cough and shortness of breath. She had been receiving antitubercular therapy for 5 months for pulmonary tuberculosis. Computed tomography angiography (CTA) revealed a massive left hemothorax (Figure 1, Panel C) with a pseudoaneurysm arising from the left IPA (Figure 1, Panel A, B, D). Mass effect was seen in the form of contralateral mediastinal shift and complete collapse of the left lower lobe. The treatment options were either endovascular management or surgical repair. Surgical repair was deferred due to clinical stability associated with greater invasiveness and a favorable anatomy for embolization.

**Figure 2:**
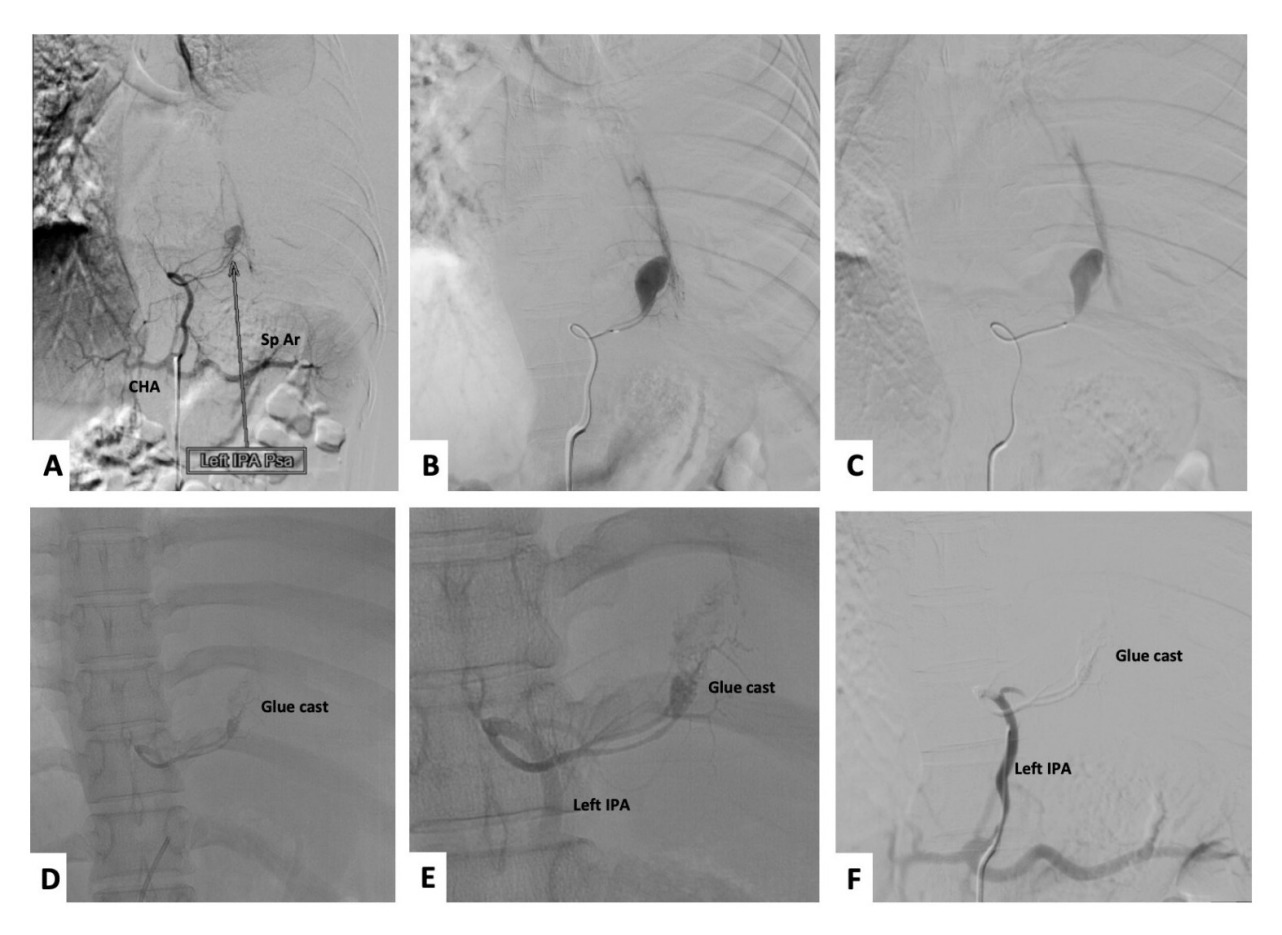
Panel A shows a selective celiac angiogram with opacification of the left inferior phrenic artery (Left IPA), common hepatic artery (CHA), and splenic artery (Sp Ar). Further microcatheter was advanced and wedged into the Left inferior division of the Left IPA (Panel B, C). Selective embolisation of the pseudoaneurysm and the descending division of the left IPA was done using a glue: lipiodol mixture (1:5 ratio) (Panels D and E). A post-embolization run revealed the patient’s left IPA with complete exclusion of the pseudoaneurysm sac (Panel F).

**Figure 3:**
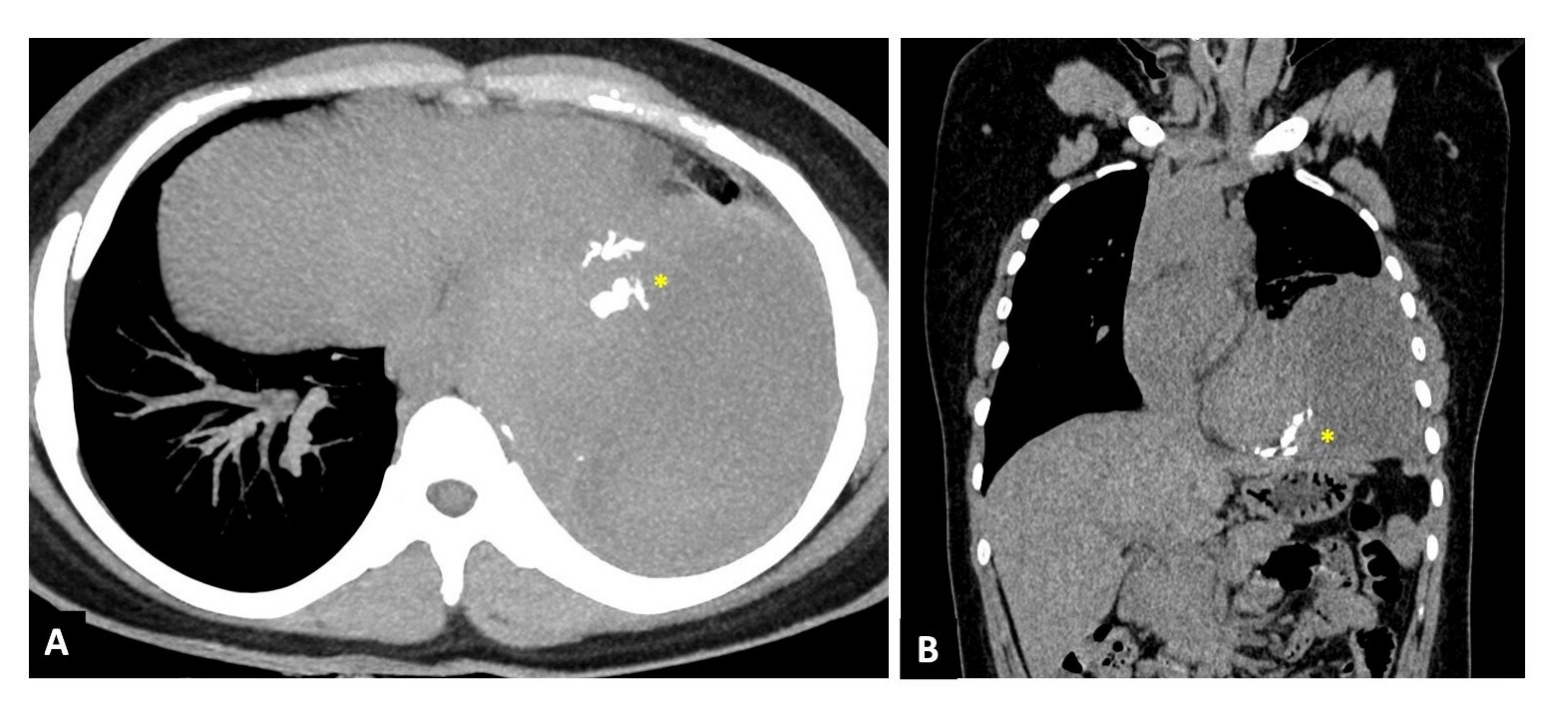
Non-contrast computed tomography (NCCT) axial in maximum intensity projection image (Panel A) and coronal image (Panel B) show glue cast in the pseudoaneurysm sac and inferior phrenic artery (Yellow asterisk) with large surrounding hematoma.

Informed consent was obtained from the patient. Institutional ethical approval was waived for this case report. Right transfemoral arterial access was obtained with a 6F femoral sheath. A diagnostic run of the celiac trunk revealed a pseudoaneurysm arising from the descending division of the left inferior phrenic artery (Figure 2, Panel A, B, C), which was successfully embolized with an N-butyl cyanoacrylate glue: lipiodol mixture (mixture ratio-1:5) (Figures 2D and 2E). A post-embolization check run revealed complete exclusion of the IPA pseudoaneurysm sac (Figure 2F). The follow up CT scan at 1 week revealed complete exclusion of the pseudoaneurysm sac with glue cast in the pseudoaneurysm sac and the left IPA (yellow asterisk, Figure 3). Subsequent hematoma evacuation was done. She was asymptomatic at 2-month follow-up.

## DISCUSSION

IPA pseudoaneurysms are extremely rare vascular pathologies with etiologies being iatrogenic,^2^ trauma, and sequelae to pancreatitis.^3^ Spontaneous IPA pseudoaneurysms are rarely described as a cause of massive hemothorax, except for one case report.^1^ The IPA typically arises from the abdominal aorta or the celiac trunk (as in our index case). Given the vessel’s deep anatomical location and small size, IPA pseudoaneurysms are often difficult to diagnose early and may present with non-specific clinical features. However, imaging through computed tomography pulmonary angiography (CTPA) and subsequent confirmation via digital subtraction angiography (DSA) identified a large pseudoaneurysm originating from the left IPA. Management of IPA pseudoaneurysms was traditionally surgical, resulting in elevated morbidity and fatality rates. Advances in imaging technology and interventional percutaneous treatments have led to prevalent use of endovascular percutaneous therapy for patient treatment.^1-5^ Endovascular embolization has minimal morbidity and mortality rates, with effectiveness ranging from 70% to 100%.^4^ These pseudoaneurysms are now predominantly treated using endovascular embolization instead of open repair, reportedly due to fewer complications and better outcomes.^5^

In conclusion, IPA pseudoaneurysms are rare, fatal vascular pathologies that can mimic infectious pathology and pose a significant risk due to potential rupture. Endovascular embolization offers a minimally invasive, effective, and lower-risk treatment option.

## Data Availability

Data not reported or used: “Data sharing does not apply to this article, as no data were generated or analyzed.”
